# Inhibitory Mechanism of an Allosteric Antibody Targeting the Glucagon Receptor*[Fn FN2]

**DOI:** 10.1074/jbc.M113.496984

**Published:** 2013-11-04

**Authors:** Susmith Mukund, Yonglei Shang, Holly J. Clarke, Azadeh Madjidi, Jacob E. Corn, Lance Kates, Ganesh Kolumam, Vicky Chiang, Elizabeth Luis, Jeremy Murray, Yingnan Zhang, Isidro Hötzel, Christopher M. Koth, Bernard B. Allan

**Affiliations:** From the Departments of ‡ Structural Biology,; § Antibody Engineering,; ¶ Molecular Biology,; ** Biomedical Imaging,; ‡‡ Protein Chemistry, and; ‖ Early Discovery Biochemistry, Genentech, Inc., South San Francisco, California 94080

**Keywords:** Antibody Engineering, Diabetes, G Protein-coupled Receptors (GPCR), Glucose Metabolism, Structural Biology

## Abstract

Elevated glucagon levels and increased hepatic glucagon receptor (GCGR) signaling contribute to hyperglycemia in type 2 diabetes. We have identified a monoclonal antibody that inhibits GCGR, a class B G-protein coupled receptor (GPCR), through a unique allosteric mechanism. Receptor inhibition is mediated by the binding of this antibody to two distinct sites that lie outside of the glucagon binding cleft. One site consists of a patch of residues that are surface-exposed on the face of the extracellular domain (ECD) opposite the ligand-binding cleft, whereas the second binding site consists of residues in the αA helix of the ECD. A docking model suggests that the antibody does not occlude the ligand-binding cleft. We solved the crystal structure of GCGR ECD containing a naturally occurring G40S mutation and found a shift in the register of the αA helix that prevents antibody binding. We also found that alterations in the αA helix impact the normal function of GCGR. We present a model for the allosteric inhibition of GCGR by a monoclonal antibody that may form the basis for the development of allosteric modulators for the treatment of diabetes and other class B GPCR-related diseases.

## Teaser

**Background:** Allosteric regulators of GPCRs provide unique pharmacological properties.

**Results:** The mechanism of allosteric inhibition of the glucagon receptor by an antibody, which is uniquely sensitive to a naturally occurring G40S mutation, is detailed.

**Conclusion:** Allosteric sites on the glucagon receptor extracellular domain regulate receptor activity.

**Significance:** Mechanisms of allosteric regulation of GPCRs aid discovery of drugs with improved selectivity.

## Introduction

Members of the class B family of GPCRs^3^ mediate the activity of peptide hormones that control many physiological functions including glucose metabolism, calcium homeostasis, vasodilation, and nociception. Numerous biochemical and structural studies show that for most of these receptors, the ECD forms a shallow, hydrophobic cleft that binds the carboxyl-terminal portion of the peptide ligand while the amino-terminal half of the ligand binds to the juxtamembrane domain of the receptor (1–5). Based on studies with the glucagon receptor, we recently proposed a model of receptor activation in which the ECD not only binds and presents glucagon to the transmembrane core for receptor activation but also undergoes a conformational change upon ligand binding that relieves inhibition of the receptor by the ECD. This negative regulation of the receptor by the ECD is mediated by an interaction between the ECD and extracellular loop 3 of the transmembrane α-helical bundle, an activity uncovered through the characterization of an inverse agonist of GCGR (6). These studies highlighted the potential for regulating the activity of class B GPCRs through novel mechanisms by targeting their ECDs.

The ligand-binding cleft in the ECD is the target of small molecule, peptide, and antibody antagonists for receptors in the class B family. For example, calcitonin gene-related peptide receptor antagonists occlude the hormone-binding cleft of the receptor (7), and our previous studies have shown that the GCGR antagonist antibodies mAb1 and mAb23 block the hormone-binding cleft of GCGR to prevent glucagon binding (6). Similarly, an antagonist antibody of the glucose-dependent insulinotropic polypeptide receptor interacts with residues in the glucose-dependent insulinotropic polypeptide-binding cleft of the ECD (8). It is also likely that there are competitive antagonists of class B GPCRs that target the hormone-binding site in the transmembrane region of these receptors (9, 10). Much less is known about the receptor sites responsible for allosteric regulation of class B GPCRs, particularly through interactions with their ECDs (11). This is an important avenue for research as the identification of novel, non-orthosteric sites that can modulate GPCR activity has the potential to yield therapeutics with increased target specificity and pathway selectivity, which in turn can provide greater potency and safety (12, 13).

Here, we show that an inhibitory monoclonal antibody, mAb7, inhibits GCGR through an allosteric mechanism as it binds to regions of the ECD outside the hormone-binding cleft. Unlike the mAb1 and mAb23 antagonists that interact with residues critical for glucagon binding, mAb7 is not dependent on residues in the binding cleft for its inhibitory activity (6). We found that mAb7 interacts with the amino-terminal αA helix of the ECD, as well as with residues on the opposite face of the ECD to the glucagon-binding cleft. Biochemical and structural studies of a naturally occurring G40S mutant receptor, which is resistant to mAb7 inhibition, point to a role for the αA helix in mediating conformational changes in the ECD that can influence receptor activity. The data presented here provide a rationale for blocking the activity of a class B GPCR through an allosteric mechanism.

## EXPERIMENTAL PROCEDURES

### 

#### 

##### Antibodies, Recombinant Proteins, and Assays

Identification of mAb7 and production of recombinant antibodies and ECD protein were described previously (6). CRE-luciferase and Quantigene bRNA (Affimetrix) assays were used to measure GCGR activity in cells (6). A competition Alphascreen assay (PerkinElmer Life Sciences, Waltham, MA) was used to monitor the ability of soluble WT or G40S GCGR ECD to compete with wild-type GCGR ECD on donor beads for binding to mAb7 and its variants on acceptor beads, as described (6). EC_50_ and IC_50_ calculations were performed using PRISM Graphpad (version 6.0) for each antibody and each glucagon-induced gene independently. The fold induction values, determined from triplicates for each glucagon or antibody concentration, were calculated in Microsoft Excel, transferred to PRISM, and transformed to log values. Non-linear regression analysis was then performed on the transformed data. For EC_50_, we used log(agonist) *versus* response; variable slope. For IC_50_, we compared the models of “log(inhibitor) *versus* response − variable slope (four parameter)” to “log (inhibitor) *versus* response (3 parameter)” or to “log(inhibitor) *versus* normalized response” using Akaike's informative criteria comparison method to select the model that most likely generated the data. The model that was determined to be the best was then used to fit the curve and establish the IC_50_ value and 95% confidence interval.

##### Shotgun Alanine Scanning of GCGR ECD

*Escherichia coli* were co-infected with a phagemid (pS2202b) (14) that was modified to contain human GCGR ECD (Ala-26 to Gln-142) and M13-KO7 helper phage, to generate M13 bacteriophage particles displaying the maltose-binding protein secretion signal, followed by an epitope tag (amino acid sequence, SMADPNRFRGKDLGS), followed by GCGR ECD and ending with the mature M13 gene-8 major coat protein on the surface. Libraries, containing ∼10^10^ unique members, were constructed and phages from the libraries were propagated in *E. coli* XL1-blue using methods described previously (15). For each mutated position, the codon was designed to encode either wild-type or alanine. For some residues, two other extra mutations might be introduced (16). Phage solutions (10^12^ phage/ml) were added to BSA-blocked, 96-well Maxisorp immunoplates that had been coated with capture mAb. For the display selection, an antibody that recognized the epitope tag fused to the N terminus of GCGR ECD was used, whereas for the functional selection, mAb7 was used. Individual clones from the fourth round of selection were screened with spot phage ELISA. Clones exhibiting signals at least 2-fold greater than signals on control plates coated with BSA were considered positive. These positive clones were subjected to DNA sequence analysis. ∼100 positive clones were sequenced for each library. The ratio, called the F value, of the number of clones recovered by mAb7 and the epitope tag mAb were calculated for each position as described previously (16).

##### Engineering and Affinity Maturation of mAb7

Humanization of mAb7 to mAb7.v1 was performed as described previously (17). The variable regions of mAb7.v1 were cloned into a previously described Fab phage display vector (18). Affinity maturation was performed by scanning mutagenesis of the heavy and light chains by phage display to identify favorable mutations (19). Two clones were produced, in each of which the three complementarity determining regions (CDRs) of the heavy or light chains were replaced by stop codons. Phage libraries were made by repairing the three CDRs of each chain with randomized oligonucleotides by oligonucleotide-directed site mutagenesis as described previously (19). For selection with human GCGR, phage libraries were incubated with biotinylated human GCGR ECD (1 nm) for 30 min followed by adding mAb7.v1 (1 μm) for 1 h to compete lower affinity binders. The GCGR ECD in the mixture was captured in streptavidin-coated plates, washed with PBS/0.1% Tween 20 and phage were eluted in 10 mm HCl, neutralized with 1/12 volume of Tris base, and used for amplification in *E. coli* XL1-Blue and additional rounds of selection. For selection with murine GCGR ECD, biotinylated antigen was immobilized on streptavidin-coated plates, incubated with phage libraries for 1 h, washed, eluted, and amplified as above. Clones from the third and fourth rounds of selection were sequenced, and preferred mutations were tabulated. Mutations identified in the humanized antibody background were introduced into the murine mAb7 clones by oligonucleotide-directed site mutagenesis.

##### Mouse Experiments

The protocols for animal experiments were approved by the Genentech Institutional Animal Care and Use Committee. Mice were maintained in a pathogen-free animal facility at 21 °C under standard 12-h light/12-h dark cycle with access to a standard rodent chow and water *ad libitum*. Male db/db mice on BKS background were purchased from The Jackson Laboratory. mAb7.v35 in PBS was injected i.p. at a dose of 2 mg/kg. Fed blood glucose levels in 10 μl of blood drawn from the tail vein were measured using a One Touch Ultra glucometer. Glucose tolerance tests were performed 4 days after dosing, as described (20). Briefly, 0.5 g/kg of glucose was injected into mice by i.p. injection after an overnight fast. Blood glucose was measured in 10 μl of blood at regular intervals after glucose administration.

##### Crystallization and Data Collection

Sitting drop vapor diffusion crystal trials of Fab fragments and GCGR G40S ECD·mAb1 complex were performed at 4 and 19 °C using a crystallization robot (Mosquito, TTP LabTech, Inc., Cambridge, MA), with drop volumes of 0.1 μl of protein sample mixed with 0.1 μl of well solution. Hit optimization was by hanging drop vapor diffusion in 24-well screw-cap plates (Qiagen, Inc., Valencia, CA). mAb7 Fab crystals grew in 0.1 m HEPES, pH 7.0, 30.0% Jeffamine ED-2001 (v/v) pH 7.0 at 15 mg/ml. Crystals appeared after 3 days as ∼25 × 25 μm thin plates. Crystals were frozen in mother liquor with 10% glycerol prior to data collection. The mAb1 Fab·GCGR G40S ECD complex was crystallized as described previously for the WT ECD·mAb1 Fab complex (6). Data were collected at the Advanced Light Source BL5.0.1 (GCGR-ECD·mAb1 complex) and BL5.0.2 (mAb7 Fab) beamlines.

##### Structure Determination and Refinement

The structure of mAb7.v16 solved by molecular replacement using Fc and Fv regions of Protein Data Bank codes 1FVC and 1FVD as the search models. Clear *F_o_* − *F_c_* electron density was observed for the GCGR G40S ECD, and this was rebuilt and refined using Coot (21) and PHENIX (supplemental Table S1) (22). Ramachandran statistics for mAb1/G40S ECD were as follows: Ramachandran outliers were 0.37 and 5% in the favored regions; and for mAb7, Ramachandran outliers were 0 and 98% in the favored regions.

##### Molecular Dynamics

Molecular dynamics simulations of WT and G40S GCGR ECD were performed using GROMACS (23). The crystal structures of “apo” WT (Protein Data Bank code 4ERS) and G40S mutant (Protein Data Bank code 4LF3) ECD were prepared by removing mAb1 and replacing any missing side chains with the most populated rotamer without clashes. Simulations were performed with the AMBER99sb force field, explicit solvent (TIP3P and 150 mm NaCl), and Particle Mesh Ewald electrostatics in a dodecahedral box with periodic boundary conditions. Starting configurations were energy minimized in vacuum for 500 steps, solvated, then reminimized for another 500 steps. Minimized coordinates were equilibrated with constrained bond lengths for 50 ps (2-fs time steps) at 200 K and then fully equilibrated without constraints for 100 ps each in successive moles (N), volume (V), temperature (T) and moles (N), pressure (P), temperature (T) simulations (1 bar, 300 K or 310 K). Production simulations were performed with constant pressure and temperature (1 bar, 300 K or 310 K). Three independent simulations were initiated for each starting structure and at each temperature by assigning random velocities at the beginning of the moles (N), pressure (P) and temperature (T) equilibration. Full input files for all steps of molecular dynamics are available in the supplemental data.

##### Computational Docking and Modeling

**FIGURE 1.**
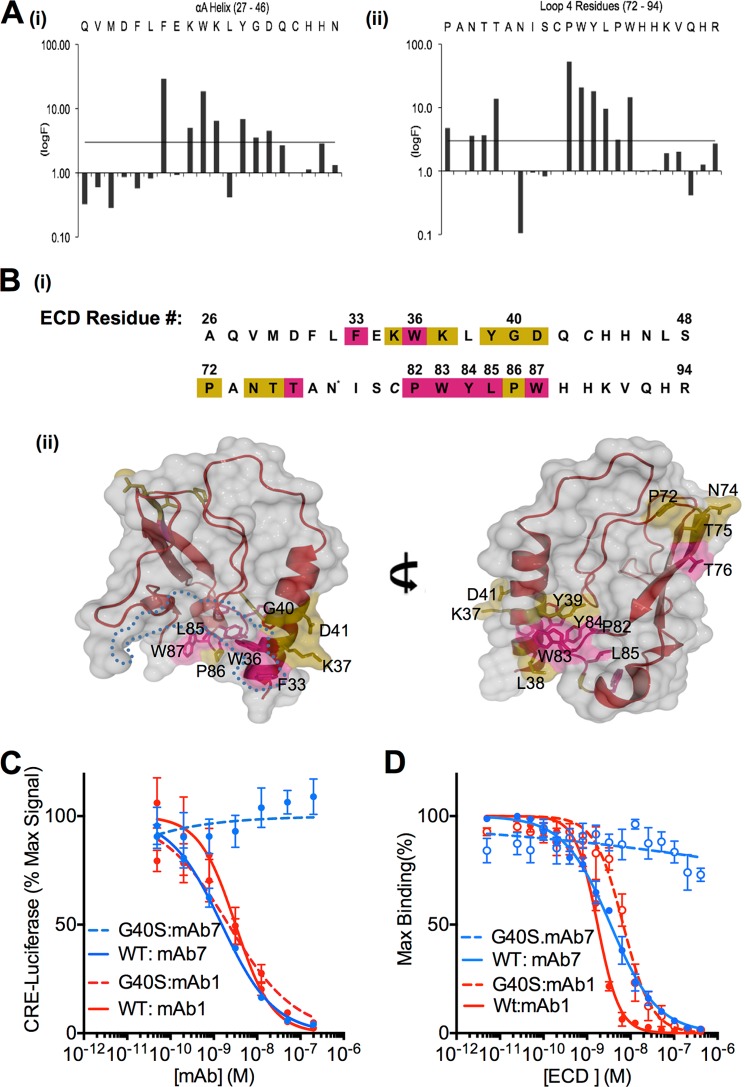
**mAb7 binds the ECD and inhibits WT but not G40S GCGR activation.** *A*, mapping of amino acids required for mAb7 binding by alanine-scanning mutagenesis of GCGR ECD. F values (21) for individual amino acids in the αA helix (residues 27–46 (*i*)) and L4 (residues 72–94 (*ii*)) are graphed. The *horizontal line* represents the cutoff for F values (logF > 3) considered meaningful using this method. *B*, *i*, linear epitope map for mAb7 interactions with the ECD. Residues that have a calculated F values between 3 and 5 or >5 are labeled in *wheat* or *magenta*, respectively. *, phage libraries held Ala constant at this position; the natural residue at this position (Asp) is shown. *ii*, GCGR alanine mutations that impact mAb7 binding are mapped onto the surface of the GCGR ECD, colored as described in *i* and labeled. The boundary of the predicted glucagon-binding cleft (6) is highlighted with *blue dots. C*, mAb7 (*blue*) fails to inhibit glucagon-induced activation of full-length, human, G40S GCGR in 293 cell-based CRE-luciferase assays. Inhibition of GCGR by mAb1 (*red*) is shown as a control. Data are mean ± S.E. (*n* = 3 independent experiments). *D*, Alphascreen competition assay measuring the ability of soluble WT or G40S GCGR ECD to compete with WT ECD bound to donor beads for binding to mAb7 (*blue*) or mAb1 (*red*) on acceptor beads. Data are mean ± S.E. (*n* = 3 independent experiments).

Experimentally guided computational docking was performed using HADDOCK (version 2.0) (24). The crystal structures of mAb7 and the G40S mutant ECD were used as the starting models. Ambiguous interaction restraints were derived from all Ala scan mutagenesis data of the ECD (as shown in Fig. 1*a*) and affinity maturation data for mAb7 on human GCGR. All active residues were selected based on being ambiguous interaction restraints and having >50% solvent accessibility. Passive residues were selected based on being solvent accessible surface neighbors of the active residues. An ensemble of 200 structures, obtained after automated refinement, was clustered into 10 clusters using backbone root mean square deviation. Clusters 1 and 2 contained the largest number of poses, 26 and 15% respectively. The docking pose with the lowest energy HADDOCK score from cluster 1 was selected for further analysis (*i.e.* representing the best HADDOCK model).

## RESULTS

### 

#### 

##### The GCGR Antagonist Antibody mAb7 Targets Two Distinct Sites on the ECD

We previously described a series of monoclonal antagonist antibodies, including an antibody called mAb7, which interact exclusively with the GCGR ECD, inhibit glucagon binding, and block GCGR activation (6). To identify the GCGR binding epitopes of these antibodies, alanine scanning mutagenesis was performed in which libraries of ECD point mutants were displayed on the surface of phage and screened for binding to antibodies in solution (6, 15). We mapped clusters of amino acids required for mAb7 binding to three distinct regions of the ECD (Fig. 1, *A* and *B*). The first cluster comprised residues Pro-82, Trp-83, Tyr-84, Leu-85, and Trp-87 on loop 4 (L4). Notably, residues Pro-82 and Trp-83 are surface exposed only on the face of the ECD opposite the glucagon binding cleft, whereas Tyr-84, Leu-85, and Trp-87 are surface-exposed on both faces of the ECD (Fig. 1*B*, *ii*). These mutations do not perturb the overall ECD structure because binding of a different GCGR antagonist, mAb23, was unaffected by the same alanine substitutions (6). In addition, another antagonist, mAb1, which completely occludes the ligand binding cleft on GCGR, required Tyr-84, Leu-85, and Trp-87, but not Pro-82 and Trp-83 for binding (6), suggesting that mAb7 may bind an epitope that is outside the glucagon-binding cleft. The second cluster of amino acids required for mAb7 binding comprised several residues in the amino-terminal αA helix of GCGR. Alanine substitution of Phe-33 or any residue from Lys-35 through Gln-42, other than Leu-38, resulted in significant loss of mAb7 binding (Fig. 1*A*, *i*). The mAb1 and mAb23 antibodies can bind to ECD containing an alanine substitution at any residue in the αA helix (6), indicating that these mutations do not significantly disrupt the overall structure of the ECD and that this epitope is unique to mAb7. The final cluster of residues on the ECD that was required for binding to mAb7 is located on L3 and comprises residues Pro-72 and Asn-74 to Thr-76 (Fig. 1*A*, *ii*, and 1B, *ii*, *right panel*). However, it is likely that these residues are important for maintaining the overall structure of the ECD and do not directly contact mAb7 because mAb1 is similarly sensitive to a T75A mutation even though this residue is not in direct contact with the antibody in the mAb1:ECD co-crystal structure (6).

Although a number of amino acids within the αA helix are required for mAb7 binding, we focused further experiments on the glycine residue at position 40 because a natural variant, S40, has been found with increased frequency in some patients with diabetes or hypertension (25–27). Carriers of this G40S mutation display a reduced response to exogenous glucagon (28). We found that mAb7 was unable to block glucagon-induced activation of G40S GCGR in a cell-based assay, whereas the previously characterized inhibitory antibody mAb1 (6, 29, 30) blocked the mutant receptor with potency equivalent to WT (wild-type) receptor (Fig. 1*C*). Interestingly, rodent GCGR contains a serine residue at position 40 and mAb7, which was generated in mice, failed to block glucagon activation of the mouse receptor (see Fig. 4*D*). We also compared the ability of mAb7 to bind to recombinant WT or G40S ECD in solution. Consistent with the results from alanine scanning mutagenesis, soluble G40S ECD failed to compete with WT GCGR for binding to mAb7, whereas WT and G40S ECDs compete for mAb1 binding (Fig. 1*D*).

##### The G40S Mutation Alters GCGR Activity and Protease Sensitivity

**FIGURE 2.**
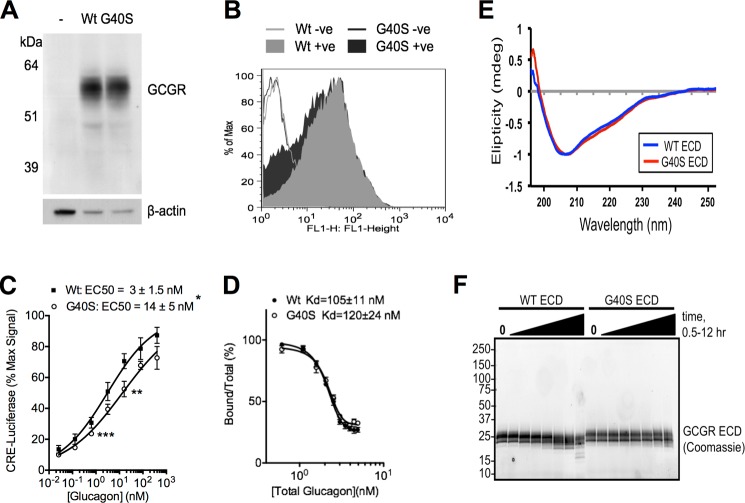
**The G40S mutation in human GCGR alters receptor conformation and function.** *A*, Western blot of stable 293 cells expressing WT or G40S GCGR, probed with anti-GCGR mAb1. *B*, FACS of stable 293 cells expressing WT or G40S GCGR using anti-GCGR mAb1. WT or *G40S -ve* refers to cells incubated without anti-GCGR. *C*, glucagon-induced activation of G40S GCGR is reduced compared with WT GCGR in CRE-luciferase assays in stable 293 cells. Data are mean ± S.E. (*n* = 3 independent experiments). The IC_50_ values are calculated from the data shown and are presented ± 95% confidence intervals. *, *p* = 0.02 and **, *p* = 0.06 (*t* test) for receptor activation at individual glucagon concentrations (*n* = 3). *D*, ^125^I-glucagon binds to WT and G40S GCGR expressed in 293 cells with comparable affinities. Data are mean ± S.E. (*n* = 3 independent experiments). *E*, CD spectra showing the αA-helix in G40S ECD is intact and indistinguishable from WT ECD. *F*, G40S ECD shows increased resistance to AspN protease digestion compared with WT Coomassie staining of recombinant ECDs after AspN digestion for up to 12 h.

To gain further insight into the role of Gly-40 in ligand binding, receptor activation, and mAb7-mediated inhibition, we tested the ability of glucagon to bind to and activate the G40S receptor in cell-based assays. In stable cells expressing equal amounts of WT and G40S GCGR on their cell surface (Fig. 2, *A* and *B*), we found that glucagon-induced activation of the G40S GCGR was reduced 4-fold, with the EC_50_ for glucagon activation increasing from 3 ± 1.5 nm for WT receptor to 14 ± 4.5 nm for G40S GCGR (Fig. 2*C*). However, we found no detectable difference in the affinity of ^125^I-glucagon for G40S compared with the WT receptor (Fig. 2*D*), suggesting that the G40S mutation reduces glucagon-induced receptor activation without impairing ligand binding. To determine whether the G40S mutation caused a conformational change in the ECD, we compared the CD spectra of purified recombinant ECDs of the two variants. The spectra overlap well, indicating that the G40S mutation does not significantly alter the secondary structure of the ECD (Fig. 2*E*). This is not unexpected because three disulfide bonds stabilize the overall ECD structure (6), and mAb1 retains potent antagonist activity on G40S receptor (Fig. 1*C*). However, we found that in contrast to WT ECD, recombinant G40S ECD was resistant to cleavage by the protease AspN (Fig. 2*F*). Taken together, these results suggest that the GCGR G40S mutation may alter the conformation of the ECD, resulting in a modest reduction in receptor activation and complete abrogation of mAb7 binding.

##### Crystal Structure of GCGR G40S ECD

**FIGURE 3.**
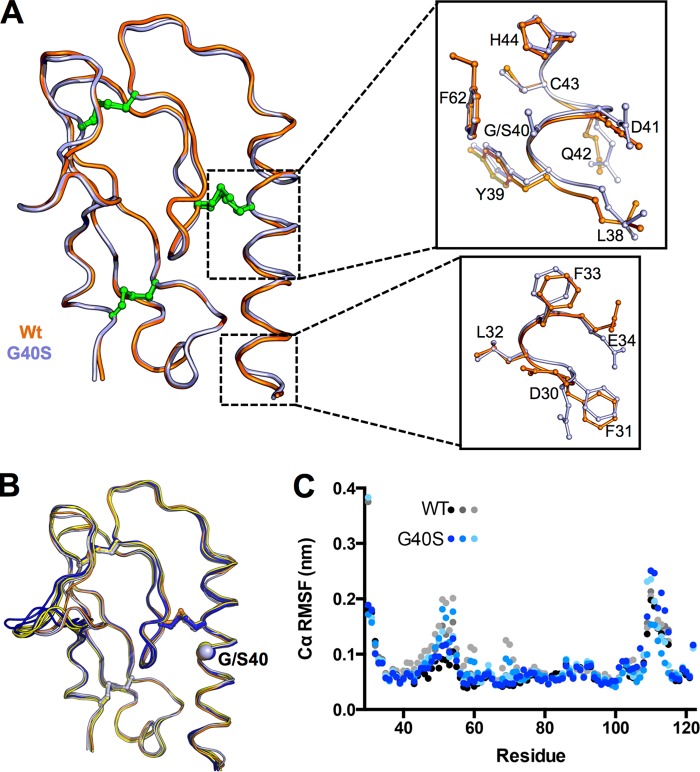
**Crystal structure and dynamics of the G40S ECD.** *A*, ribbon representation of GCGR WT and G40S ECD structures illustrates a shift in the αA helix but overall high structural similarity. *Top inset*, comparison of WT and G40S structures around residue 40. *Bottom inset*, comparison of WT and G40S structures in the region of residues 30–34, highlighting observed orientations of the amino acid side chains. Disulfide bonds are *green. B*, time-averaged structures over three independent 100 ns molecular dynamics simulations of the WT and G40S ECDs at 300 K are very similar to the starting structures. The WT and G40S starting structures are shown in *orange* and *gray*, respectively. Averaged structures for WT and G40S molecular dynamics simulations are shown in *blue* and *yellow*, respectively. Disulfide bonds are shown as sticks, and the Cα of position 40 is shown as a *sphere* for reference. *C*, the root mean square fluctuation (*RMSF*) of Cα atoms for three each independent molecular dynamics simulations of the WT and G40S ECD highlight nearly identical flexibility for each protein.

To define at the atomic level the effect of G40S on the conformation of the αA helix and the ECD, we solved the crystal structure of the G40S ECD and compared it to the structure of the WT ECD we described previously (Fig. 3) (6). As with WT ECD, the G40S mutant could only be crystallized as a complex with the monoclonal antibody mAb1. We have yet to find conditions in which the ECD can crystallize alone. Crystals of the G40S ECD·mAb1 complex diffracted x-rays to 2.88 Å and are isomorphous with WT ECD/mAb1 crystals obtained in the same condition (Table S1) (6). The wild-type and G40S structures are extremely similar, with a Cα root mean square deviation over 126 residues of 0.3 Å, indicating that there are no major structural differences as a result of the G40S mutation. We cannot rule out that this structural similarity is at least in part a consequence of co-crystallizing with mAb1. Nevertheless, the CD spectra demonstrate that the ECDs are also structurally similar in solution in the absence of mAb1 (Fig. 2*E*). To investigate the effects of the mutation upon the flexibility of the ECD, we performed multiple molecular dynamics simulations of WT and G40S ECD in the absence of mAb1 at both room temperature and 37 °C. Apart from the relatively unrestrained loop between Trp-106 and Arg-116, both proteins retain the conformation observed in the mAb1 co-crystal structures (Fig. 3*B* and supplemental Fig. S1). Additionally, the root mean square fluctuation profiles of the two proteins are virtually identical (Fig. 3*C* and supplemental Fig. S1), indicating that the G40S mutation has a negligible effect upon the flexibility of ECD at the time scales measured here. Taken together, these data suggest that only minor structural differences between the WT and G40S ECDs must account for the biochemically detectable differences in receptor activation, protease sensitivity, and mAb7 inhibition. Comparison of the structures revealed a small shift in register along the length of the αA helix and also differences in the orientations of some side chain residues in this region, for example residues Asp-30-Glu-34 (Fig. 3).

##### Engineering mAb7 Variants to Bind and Inhibit Mouse and Human G40S GCGR

**FIGURE 4.**
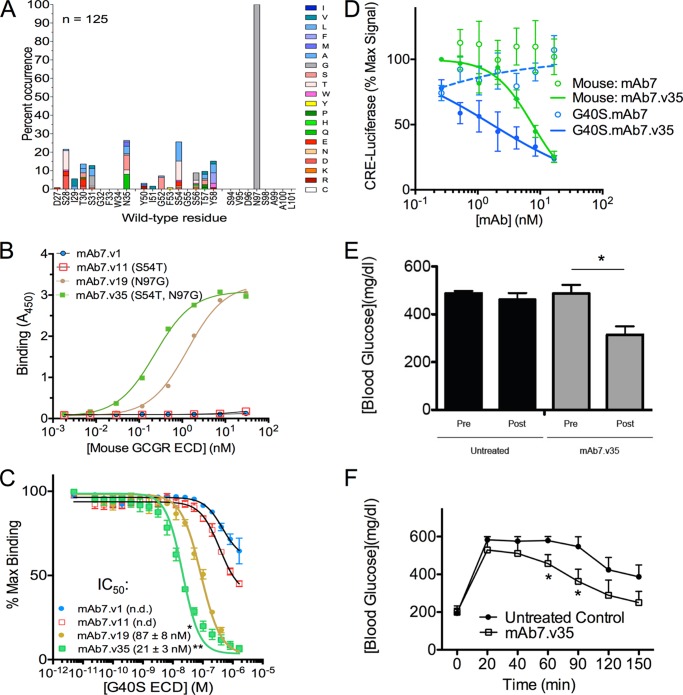
**Identification of mAb7 variants that recognize mouse and human G40S GCGR.** *A*, single site saturation mutagenesis scan for all CDR positions of mAb7 heavy chain to recombinant mouse GCGR ECD. 125 clones selected for binding to mouse GCGR ECD were sequenced to score mutations in each CDR. Each *bar* represents the percent occurrence of an amino acid other than the wild-type residue. Note that mutagenesis allows each clone to have only one mutation in each CDR. The *colors* in each bar represent individual amino acids, as indicated in the *key* on the *right* of the graph. *B*, binding of select mAb7 IgG variants identified in *A*. mAb7.v1 is a humanized version of the mouse monoclonal parental antibody mAb7. mAb7.v11, mAb7.v19, and mAb7.v35 are humanized antibodies containing S54T, N97G, or S54T/N97G mutations respectively. *C*, Alphascreen competition assay measuring the ability of soluble G40S ECD to compete with WT ECD bound to donor beads for binding to mAb7.v1, mAb7.v11, mAb7.v19, or mAb7.v35 IgG bound to acceptor beads. Data are mean ± S.E. (*n* = 3 independent experiments). The IC_50_ values were calculated from the fitted curve shown (*p* < 0.001, two-way analysis of variance; *, *versus* mAb7.v1, **, *versus* mAb7.v19 (n.d., not determined). *D*, mAb7.v35, but not mAb7, inhibits glucagon-induced activation of mouse GCGR and human G40S GCGR expressed in cells. Data are mean ± S.E. (*n* = 3 independent experiments). A single injection of mAb7.v35 (2 mg/kg) into db/db mice reduces blood glucose after 24 h (*E*) and improves glucose tolerance after 4 days (*F*). Data are mean ± S.D. (*n* = 5 mice/group, *, *p* ≤ 0.05 (*t* test)). *n.d.*, not determined.

**FIGURE 5.**
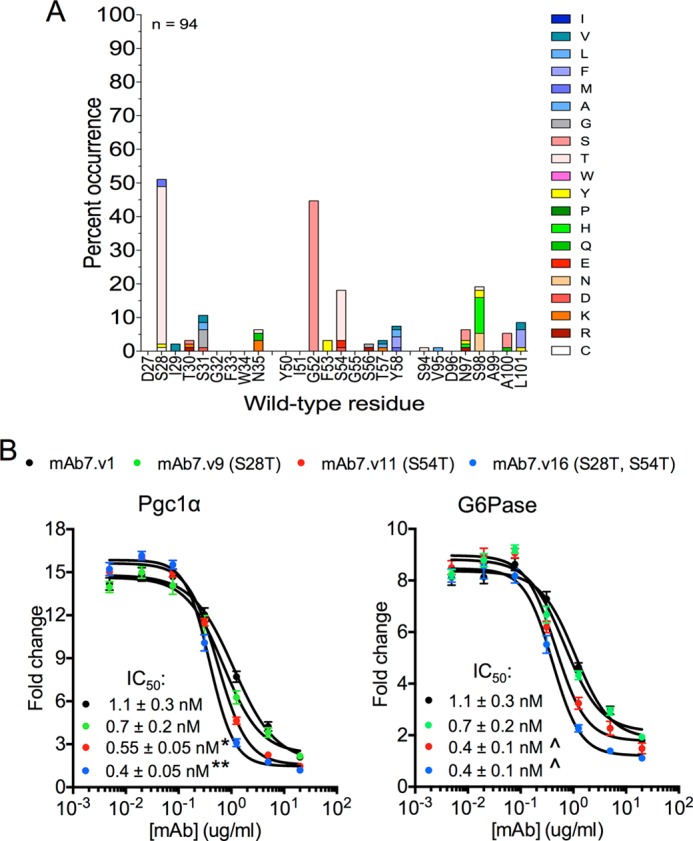
**Identification of affinity matured, humanized mAb7 variants.** *A*, single site saturation mutagenesis scan for all CDR positions of mAb7 heavy chain to recombinant human GCGR ECD. 94 clones selected for binding to human GCGR ECD were sequenced to score mutations in each CDR. Each *bar* represents the percent occurrence of an amino acid other than the wild-type residue. Note that mutagenesis allows each clone to have only one mutation in each CDR. The colors in each bar represent individual amino acids, as indicated in the key on the *right* of the graph. *B*, dose response curves of mAb7.v1, mAb7.v9 (S28T), mAb7.v11 (S54T), and mAb7.v16 (S28T, S54T), demonstrating inhibition of glucagon-induced Pgc1α and G6Pase gene expression in primary human hepatocytes. Data are mean ± S.E. (*n* = 3 independent experiments). The IC_50_ values were calculated from the data shown and are presented ± 95% confidence intervals. *, *p* < 0.05 (*versus* mAb7.v1); **, *p* < 0.001 (*versus* all other variants); , *p* < 0.001 (*versus* mAb7.v1 and mAb7.v9) using two-way analysis of variance with an uncorrected Fischer's test. Note that a G52S mutation did not improve the affinity of mAb7 when converted to IgG (data not shown).

Although the conformation of the αA helix is critical for inhibition of GCGR activity by mAb7, the structures and dynamics of the αA helix appear nearly identical between the WT and G40S mutant. We reasoned that only minor antibody engineering would be necessary to restore potency on the mouse and G40S mutant receptors. Indeed, using single site saturation mutagenesis for all CDR positions, we found that only a single point mutation, N97G in CDR H3, was required to confer binding to the mouse and human G40S mutant receptors (Fig. 4, *A–C*). An additional S54T point mutation in CDR H2 (mAb7.v35) further increased the affinity of the N97G containing antibody (mAb7.v19) for GCGR, whereas S54T alone (mAb7.v11) did not confer the ability to bind mouse or G40S (Fig. 4, *B* and *C*). The S54T mutation was also identified separately from the N97G mutation, during affinity maturation of mAb7.v1 on human WT GCGR (Fig. 5). We tested the ability of these mAb7 variants to inhibit glucagon activation of GCGR. In contrast to mAb7, mAb7.v35 was a potent inhibitor of both mouse and human G40S receptors both *in vitro* (Fig. 4*C*) and *in vivo*, as a single injection of this antibody reduced blood glucose and improved glucose tolerance in diabetic mice (Fig. 4, *D* and *E*).

##### Mechanism of mAb7 Antagonism of GCGR

**FIGURE 6.**
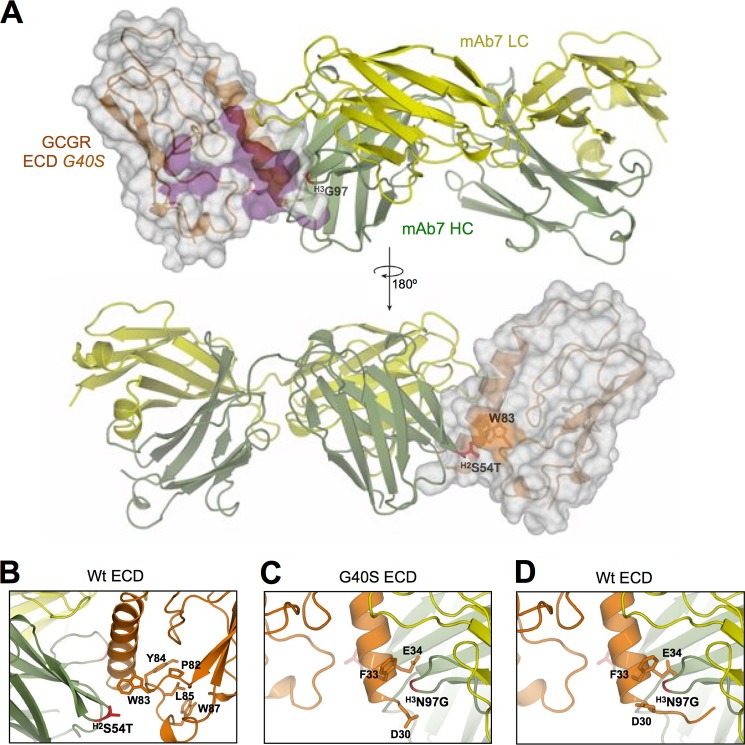
**Docking model for mAb7:ECD binding and GCGR inhibition.** *A*, a docking model of the ECD·mAb7 complex shows that mAb7 uses CDRs of the heavy chain (*green*) and light chain (*yellow*) to form a cleft that straddles the αA helix of the ECD (*orange* with surface representation). Residues on the ECD that form the glucagon-binding site as described in Ref. 6 are colored *purple*. Residues of mAb7 that yield improved binding in some variants are colored *red* and labeled; CDR locations are indicated in *superscript. B–D*, interactions between mAb7 and the GCGR ECD in the docking model. In *B*, residues on the face of the ECD opposite the ligand-binding cleft positive for mAb7 binding by alanine scan mutagenesis are indicated; affinity matured mAb7 residues are colored *red* and labeled. In *C*, ECD residues proximal to the mAb7 ^H3^G97 residue (*red*) in the G40S ECD:mAb7 docking model are labeled. The orientations of the same residues in the WT ECD structure are shown in *D*.

Next, we sought structural insight into the mechanism of mAb7 antagonism of GCGR. Despite considerable effort, we have been unable to obtain diffracting crystals of the GCGR ECD in complex with mAb7. However, we obtained crystals of the Fab fragment of a mAb7.v1 variant obtained by affinity maturation, containing S28T and S54T mutations (mAb7.v16) that diffracted to 2.0Å resolution. We found that the S54T mutation alone, or in combination with the S28T mutation, led to improved potency of glucagon-induced inhibition (Fig. 5). Using the structures of the mAb7.v16 (S28T, S54T) Fab, and of the WT and G40S ECDs, we explored the mechanism of GCGR antagonism by mAb7 by attempting to dock the Fab onto the surface of the ECD. Using HADDOCK software (24), we performed *in silico* docking studies with the G40S GCGR and mAb7 structures using the GCGR and mAb7 mutagenesis data as restraint inputs. These analyses revealed that the only reasonable orientation in which mAb7 could readily bind the ECD while being consistent with the mutagenesis data is one in which the CDRs of mAb7 largely straddled the αA helix of GCGR similar to a saddle (Fig. 6*A*). In this model, the ligand-binding cleft of the GCGR ECD is not occluded by mAb7 (Fig. 6*A*, *purple* schematic), indicating that inhibition of glucagon binding is unlikely through steric clashes between mAb7 and glucagon.

The docking model predicts that residue 54 on CDR H2 of mAb7 makes contact with the face of the GCGR ECD opposite the ligand-binding cleft (Fig. 6*B*). A threonine at this position of mAb7 improves affinity and potency on mouse and human GCGR (Figs. 4 and 5). In the docking model, this residue packs against Trp-83 of the ECD, reflecting the alanine scan mutagenesis data showing that a W83A mutation significantly reduces mAb7 binding (Fig. 1*A*). In addition, the docking model provides a probable explanation for how the N97G mutation in CDR H3 of mAb7 restores binding and activity on the mouse and human G40S mutant GCGRs. As shown in Fig. 6*C*, residue 97 in CDR H3 of mAb7 packs closely against a shallow groove formed by the side chains of residues Asp-30, Phe-33, and Glu-34 of the GCGR αA helix. In this model, an asparagine residue cannot be accommodated on mAb7 without sterically clashing with the GCGR αA helix, whereas a glycine residue could readily pack against this groove. We also note that this region of the ECD shows obvious structural differences for residues Asp-30, Phe-33, and Glu-34 when comparing the WT (Fig. 6*D*) and G40S structures (Fig. 3, *inset*). Taken together, these observations are consistent with a model in which mAb7 prevents ligand binding and receptor activation through an allosteric mechanism, through interactions with ECD residues that are surface exposed outside of the ligand-binding cleft.

## DISCUSSION

An inappropriate increase in glucagon levels drives excess hepatic glucose output and contributes to hyperglycemia in type 2 diabetes (31). We and others (6, 29, 30) have generated inhibitory monoclonal antibodies targeting GCGR that are potential therapeutics for the treatment of diabetes. All of the inhibitory antibodies that we have studied target the ECD of GCGR, but these can differ in their molecular pharmacology. For example, mAb1 is a classic competitive antagonist, whereas mAb23 is an inverse agonist (6). The mAb7 antibody is unique in that it does not require ligand-binding residues in the GCGR ECD for activity (6). To determine the molecular mechanism of mAb7 activity, we first defined the mAb7 epitope on GCGR. Residues comprising both the N-terminal αA helix and a core loop of the ECD, L4, are required for mAb7 binding. Intriguingly, some of these L4 residues are only exposed on the surface of the ECD opposite the ligand-binding cleft. Similarly, mutagenesis studies of mAb7 identified antibody residues that contribute to ECD binding and inhibitory activity. Aided by crystal structures of the ECD and mAb7, as well as the mutagenesis studies on both molecules, we generated a docking model of mAb7 bound to the GCGR ECD. In this model, CDR H2 residue 54 in mAb7 interacts with the ECD L4 residue Trp-83 on the back of the ligand binding cleft, explaining why affinity maturation efforts could yield a mutation at this position (S54T) that is favored for potent mAb7 binding and activity (Fig. 5). We propose a mechanism for mAb7 activity that relies upon disruption of the glucagon-binding cleft through interactions with L4 residues on the face of the ECD opposite the ligand-binding cleft. This would represent a classical allosteric mechanism in which ligand binding is disrupted through an interaction of the antagonist with a site outside the ligand-binding pocket.

Previously, we reported a docking model of glucagon with GCGR ECD in which glucagon interacts with amino acid side chains of the αA helix that face into the ligand binding cleft (6). Glp1 similarly interacts with αA helix residues of Glp1 receptor (5, 6, 32). We have now shown that perturbations in this region can alter receptor activation without affecting glucagon binding, defining this as an allosteric site. Specifically, on cells expressing equivalent levels of receptor, glucagon binds to G40S GCGR with equal affinity to WT GCGR but is less potent in G40S receptor activation (Fig. 2) (33). Proteolytic sensitivity and mAb7 binding experiments permitted the measurement of other distinct biochemical and structural differences between WT and G40S ECD, whereas the G40S crystal structure revealed changes in the orientations of some amino acid side chains in this region (Fig. 3*A*). Although 100-ns molecular dynamics simulations indicate very similar flexibility between the WT and G40S ECDs, we cannot rule out differences in conformation or dynamics over longer time scales. Indeed, the increased sensitivity of the WT ECD to AspN digestion is only apparent after several hours of incubation. We propose that conformational changes in the GCGR αA helix are capable of altering receptor activity, possibly through a mechanism involving interactions with other regions of the ECD and/or with the receptor membrane core. For example, we have previously described a network of interactions between Tyr-65 in the ligand binding cleft and other regions of the ECD, including the αA helix, as well as an interaction between the ECD and the receptor membrane core that regulates receptor activity (6). Interfering with these interactions via ligand or antibody binding could contribute to conformational changes in the ECD that are associated with receptor activity.

The change in register of the αA helix observed in the G40S ECD structure, although minor, probably explains the loss of binding of mAb7 to G40S ECD and the subsequent rescue of mAb7 binding by introduction of an N97G mutation in the mAb7 CDR H3. We suspect that the change in register generates a clash between Asn-97 of mAb7 CDR H3 and the αA helix residues Asp-30, Phe-33, and/or Glu-34 (Fig. 6, *C* and *D*). The substitution of the Asn-97 side chain for the smaller glycine side chain likely removes this clash thereby enabling mAb7 with a N97G mutation to bind and inhibit the G40S receptor. Indeed, the combination of this N97G mutation with the S54T mutation identified during affinity maturation (Figs. 4*A* and 5*A*) generated an antibody (mAb7.v35) that is efficacious *in vivo*, with a single injection reducing hyperglycemia and improving glucose tolerance in diabetic mice (Fig. 4, *D* and *E*).

This work broadens the repertoire of GCGR antagonists by defining mAb7 as an allosteric inhibitor. Allosteric modulation of GCGR, and other class B family GPCRs, is not unprecedented. For example, L-168,049 is a small molecule antagonist of GCGR that behaves as a non-competitive antagonist of glucagon, but unlike mAb7, it interacts with the transmembrane core of the receptor (34). In addition, a non-competitive, allosteric antagonist of the Glp1 receptor, T-0632, requires Trp-33 in the Glp1 receptor αA helix for binding and activity (35). The demonstration that mAb7 can modulate receptor activity allosterically may provide some insight into regulation of GCGR by endogenous factors that target its ECD. A role for ECD-binding proteins that regulate the activity of other class B family GPCRs has been well established. RAMP1 (receptor accessory modifying protein 1) binds to the ECD of the calcitonin receptor-like receptor to facilitate localization of calcitonin receptor-like receptor to the cell surface where the heterodimer forms the calcitonin gene-related peptide receptor. In the absence of RAMP1, calcitonin receptor-like receptor binds no known endogenous ligand. RAMP1 is one of several RAMP proteins that each confer unique ligand specificity on their GPCR partners (36, 37). More recently, interaction between lipoprotein-related protein family members Lrp5 and Lrp6 and GCGR, as well as the parathyroid hormone receptor, have been shown be required for receptor activation (38, 39). The molecular details of the interaction and the mechanism by which these interactions regulate and modify receptor activity are unknown; however, it is tempting to speculate that interactions with the ECDs of the respective proteins provide a basis for allosteric regulation of receptor activation. The identification of mAb7 as an allosteric antagonist of GCGR expands our understanding of the molecular basis for inhibition of class B GPCRs and opens up the potential for developing antibody allosteric regulators of these receptors by targeting specific regions on their ECDs.

## Footnotes

3 The abbreviations used are:

GPCR: G-protein coupled receptor
GCGR: glucagon receptor
ECD: extracellular domain
CDR: complementarity determining region.
